# Gata4 regulates hedgehog signaling and Gata6 expression for outflow tract development

**DOI:** 10.1371/journal.pgen.1007711

**Published:** 2019-05-23

**Authors:** Jielin Liu, Henghui Cheng, Menglan Xiang, Lun Zhou, Bingruo Wu, Ivan P. Moskowitz, Ke Zhang, Linglin Xie

**Affiliations:** 1 Department of Nutrition and Food Sciences, Texas A&M University, College Station, Texas, United States of America; 2 Department of Biomedical Sciences, University of North Dakota, Grand Forks, North Dakota, United States of America; 3 Tongji Hospital, Huazhong University of Science and Technology, Wuhan, Hubei, China; 4 Departments of Genetics, Pediatrics, and Medicine (Cardiology), Albert Einstein College of Medicine of Yeshiva University, Bronx, NY, United States of America; 5 Departments of Pathology and Pediatrics, The University of Chicago, Chicago, Illinois, United States of America; 6 Center for Epigenetics & Disease Prevention, Institute of Biosciences & Technology, College of Medicine, Texas A&M University, Houston, Texas, United States of America; University of North Carolina, UNITED STATES

## Abstract

Dominant mutations of Gata4, an essential cardiogenic transcription factor (TF), were known to cause outflow tract (OFT) defects in both human and mouse, but the underlying molecular mechanism was not clear. In this study, *Gata4* haploinsufficiency in mice was found to result in OFT defects including double outlet right ventricle (DORV) and ventricular septum defects (VSDs). Gata4 was shown to be required for Hedgehog (Hh)-receiving progenitors within the second heart field (SHF) for normal OFT alignment. Restored cell proliferation in the SHF by knocking-down Pten failed to rescue OFT defects, suggesting that additional cell events under Gata4 regulation is important. SHF Hh-receiving cells failed to migrate properly into the proximal OFT cushion, which is associated with abnormal EMT and cell proliferation in Gata4 haploinsufficiency. The genetic interaction of Hh signaling and Gata4 is further demonstrated to be important for OFT development. *Gata4* and *Smo* double heterozygotes displayed more severe OFT abnormalities including persistent truncus arteriosus (PTA). Restoration of Hedgehog signaling renormalized SHF cell proliferation and migration, and rescued OFT defects in *Gata4* haploinsufficiency. In addition, there was enhanced Gata6 expression in the SHF of the Gata4 heterozygotes. The Gata4-responsive repressive sites were identified within 1kbp upstream of the transcription start site of *Gata6* by both ChIP-qPCR and luciferase reporter assay. These results suggested a SHF regulatory network comprising of Gata4, Gata6 and Hh-signaling for OFT development.

## Author summary

Gata4 is an important transcription factor that regulates the development of the heart. Human possessing a single copy of Gata4 mutation display congenital heart defects (CHD), including double outlet right ventricle (DORV). DORV is an alignment problem in which both the Aorta and Pulmonary Artery originate from the right ventricle, instead of originating from the left and the right ventricles, respectively. In this study, a Gata4 mutant mouse model was used to study how Gata4 mutations cause DORV. We showed that Gata4 is required in the cardiac precursor cells for the normal alignment of the great arteries. Although Gata4 mutations inhibit the rapid increase in the cardiac precursor cell numbers, resolving this problem does not recover the normal alignment of the great arteries. It indicates that there is a migratory issue of the cardiac precursor cells as they navigate to the great arteries during development. The study further showed that a specific molecular signaling, Hh-signaling and Gata6 are responsible to the Gata4 action in the cardiac precursor cells. Importantly, over-activation of the Hh-signaling pathways rescues the DORV in the Gata4 mutant embryos. This study provides a molecular model to explain the ontogeny of a subtype of CHD.

## Introduction

Congenital Heart Defects (CHDs) occur in approximately 1% of live births [1] and are the most common serious birth defects in humans [2, 3]. Approximately one third of the CHDs involve malformations of the outflow tract (OFT), which leads to significant morbidity and mortality of children and adults [4]. Multiple OFT abnormalities involve the defective relationship between the Aorta and Pulmonary Artery to the underlying left and right ventricles. For example, double-outlet right ventricle (DORV) is an anomaly in which the Aorta and Pulmonary Artery originate from the right ventricle [4]. A key characteristic of DORV that distinguishes it from other OFT defects is that the aorta and pulmonary trunk are well separated but are improperly aligned over the right ventricle. The molecular basis of OFT misalignment has remained unclear.

SHF-derived cells migrate into the developing poles of the heart tube, to direct the morphogenesis of the cardiac inflow and outflow. The anterior SHF (aSHF) is essential for OFT and great artery development [5–9]. The failure of the aSHF-derived myocardial and endocardial contributions to the arterial pole of the heart causes a shortened OFT and arterial pole misalignment, resulting in inappropriate connections of the great arteries to the ventricular mass [10–12]. Deletion of genes responsible for SHF morphogenesis, such as *Isl1*, *Mef2c*, and *Jagged1*, leads to abnormal OFT formation including DORV [5, 6, 12–19]. These observations lay the groundwork for investigating the molecular pathways required for OFT development in SHF cardiac progenitor cells.

Gata4, a member of the GATA family of zinc finger transcription factors, is an essential cardiogenic transcriptional regulator implicated in many aspects of cardiac development and function [13–27]. Human genetic studies have implicated haploinsufficiency of GATA4 in human CHDs, such as atrial septal defects (ASD), ventral septal defects (VSD), and tetralogy of Fallot (TOF) [17, 28–32]. In mouse models, decreased expression of *Gata4* results in the development of common atrioventricular canal (CAVC), DORV, and hypoplastic ventricular myocardium in a large proportion of mouse embryos [20, 33]. Multiple studies have demonstrated the molecular requirement of Gata4 in the endocardium for normal cardiac valve formation [15, 23, 34]. Furthermore, our previous study demonstrated that *Gata4* is required in the posterior SHF (pSHF) for atrial septation. Both Hedgehog (Hh) signaling and *Pten*-mediated cell-cycle progression were shown to be downstream of *Gata4* in atrial septation [35]. However, the mechanistic requirement for Gata4 in OFT development was not clear and seemed different as in atrial septation. For example, from the multiple *Gata4* transcriptional targets that have been identified in the context of heart development, including *Nppa*, *α-MHC*, *α-CA*, *B-type natriuretic peptide* (*BNP*), *Ccnd2*, and *Cyclin D2*, *Gli1*, and *Mef2c* [13, 15, 16, 18, 36–38], only *Mef2c* has a known functional role in OFT development [12].

In this study, the mechanistic requirement for *Gata4* in OFT development was investigated. *Gata4*-dependent pathways were revealed to be contributors to OFT development in *Gata4* heterozygous mouse embryos.

## Results

### Gata4 is required for OFT alignment

**Fig 1:**
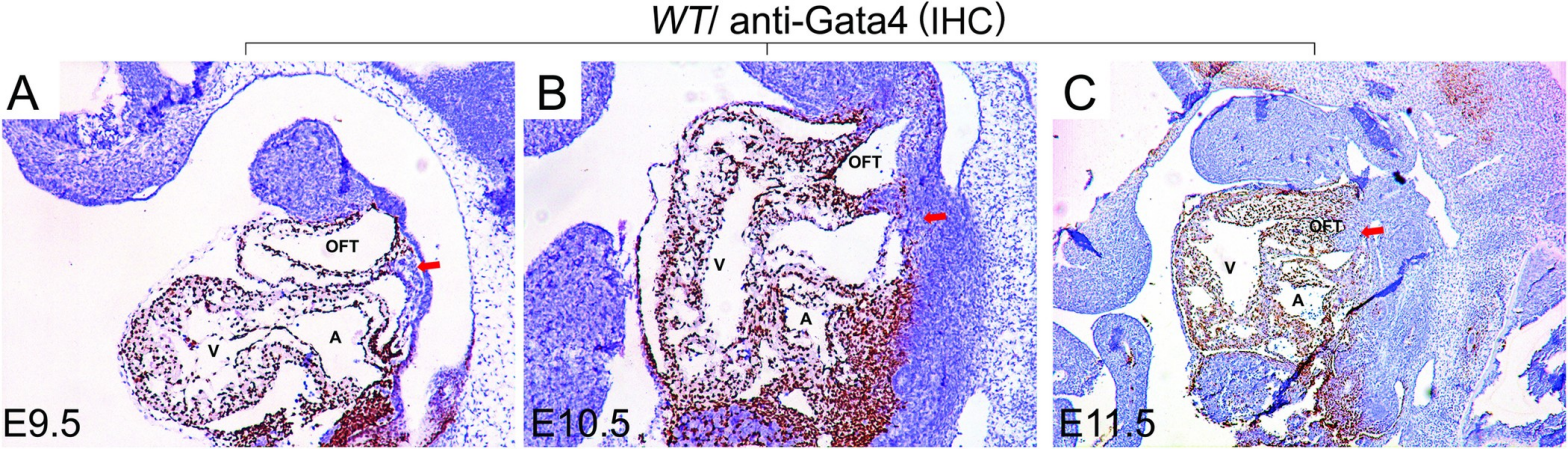
Gata4 is strongly expressed in the developing heart, the OFT and the pSHF. Gata4 expression was detected in *wildtype* mouse embryos by IHC at (A) E9.5, (B) E10.5 and (C) E11.5. Red arrows indicate anterior second heart field at E9.5 or E10.5 (A and B), and distal outflow tract at E11.5 (C). OFT: out flow tract; A: atrium; V: ventricle. Magnification: A, B and C: 100X.

*Gata4* is strongly expressed in the heart, pSHF and OFT at E9.5 [20, 35, 39]. There is a gap in expression between the OFT and the pSHF at embryonic day 9.5 (Fig 1A, indicated by a “↓”). IHC staining for Gata4 at later stages during OFT development showed strong Gata4 expression in the heart, the developing OFT and the pSHF, but only in a limited subset of aSHF cells at E10.5 (Fig 1B, indicated by a “↓”). At E11.5, both the chamber myocardium and the developing OFT had strong Gata4 expression. However, Gata4 expression was absent from the cardiac neural crest (CNC)-derived distal OFT (Fig 1C, indicated by a “↓”).

**Fig 2:**
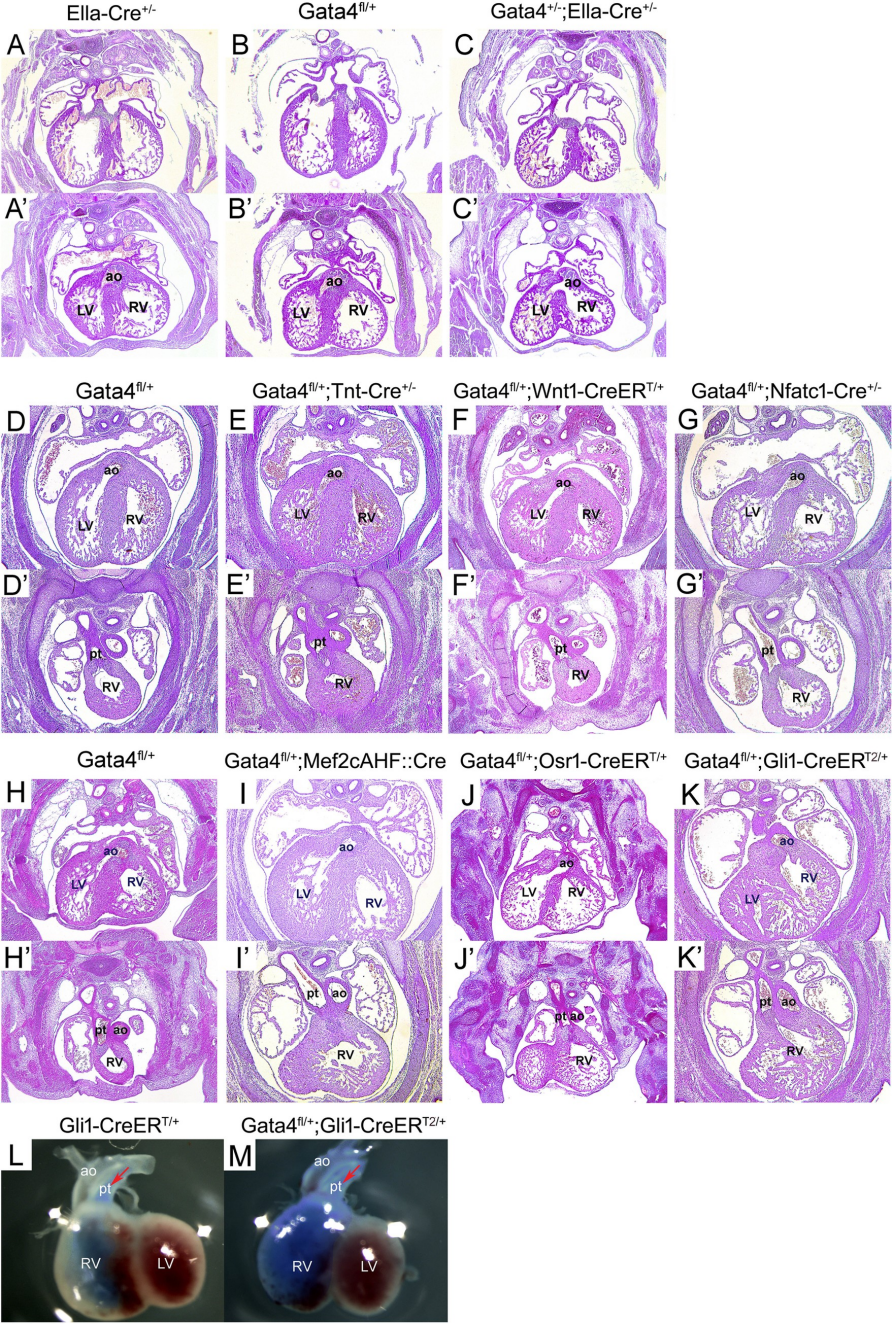
Gata4 is required in Hh-receiving cells for OFT development. (A-K’) Histology of Gata4 transgenic mouse embryonic heart at E14.5. The lower panels (A’-C’) and upper panels (D-K) showed that the opening of the AO connected to the right ventricle in the Gata4 haploinsufficency, while in control embryos the AO opened to the left ventricle. LV, left ventricle; RV, right ventricle; ao, aorta artery; pt, pulmonary trunk. Magnification: 40X (L-M) Blue ink stained embryo heart at E14.5. LV, left ventricle; RV, right ventricle; ao, aorta artery, pt, pulmonary trunk. Red arrow indicates the ink injected direction.

Gata4 was previously reported to be required for OFT alignment [20]. To study the role of Gata4 in OFT development, *Gata4* heterozygotes were examined for OFT defects. As described previously [35], *Gata4* heterozygotes were generated by crossing *Gata4*^*fl/+*^ with *Ella-Cre*^*+/-*^, which drives Cre expression in the germline [40] to produce the germline *Gata4* deletion. The Gata4 germline deletion was ensured by genotyping using the embryonic tail DNA (Fig 1S). Whereas *Gata4*^*fl/+*^ (n = 13) and *Ella*^*Cre/+*^ (n = 12) embryos demonstrated normal heart at E14.5 (Fig 2A and 2A’, 2B and 2B’), 61.1% of *Gata4*^*+/-*^; *Ella-Cre*^*+/-*^embryos demonstrated VSD and DORV (Fig 2C’, 11/18, P = 0.0004). Consistent with prior work, primum ASDs with an absence of the DMP observed in 8 out of 18 *Gata4*^*+/*^; *Ella-Cre*^*+/-*^embryos [35] (Fig 2C). In addition, 37.5% of these embryos displayed A-V cushion defects (3 out of 8, Fig 2C vs. 2A) and 62.5% expressed right ventricle hypoplasia (possibly right ventricle non-compaction) (5 out of 8, Fig 2C vs. 2A).To determine the lineage requirement for *Gata4* in AV septation, we analyzed mouse embryos haploinsufficient for *Gata4* in the myocardium, CNC, endocardium or SHF (Fig 1S). We combined *Tnt*: *Cre* [41] with *Gata4*^*fl/+*^ to create *Gata4* haploinsufficiency in the myocardium. Normal OFT alignment was observed in all *Tnt-Cre*^*+/-*^*; Gata4*^*fl/+*^ (12/12) and littermate control *Gata4*^*fl/+*^ embryos (9/9) at E14.5 (P = 1) (Fig 2E and 2E’ vs. 2D and 2D’, P = 1). *Wnt1*: *CreER*^*T/+*^ and *Gata4*^*fl/+*^ was combined to create *Gata4* haploinsufficiency in the CNC induced by tamoxifen (TMX) administration at E8.5 and E9.5 [42, 43]. Normal OFT alignment was observed in all *Wnt1-CreER*^*T/+*^*; Gata4*^*fl/+*^ mutant embryos (24/24) and littermate control *Gata4*^*fl/+*^ embryos (16/16) at E14.5 (Fig 2F and 2F’ vs. 2D and 2D’, P = 1). *Nfat1c*: *Cre* [42, 43] and *Gata4*^*fl/+*^ were combined to create *Gata4* haploinsufficiency in the endocardium. Normal OFT alignment was observed in 93.3% of the *Nfatc1-Cre*^*+/-*^*; Gata4*^*fl/+*^ mutant embryos (14/15) and all of littermate control *Gata4*^*fl/+*^ embryos (10/10) at E14.5 (Fig 2G and 2G’ vs. 2D and 2D’, P = 1). All embryos are viable at E14.5 and no other heart defects such as the atrial septal defects (ASDs), ventricle septal defects (VSDs), or malformations of the ventricular wall, were observed at this stage. These results demonstrated that *Gata4* haploinsufficiency in the myocardium, CNC or endocardium did not contribute to abnormal OFT alignment.

### *Gata4* is required in the SHF Hedgehog (Hh) signal-receiving progenitors for OFT alignment

We hypothesized that Gata4 is required in the aSHF for OFT alignment in aSHF-specific *Gata4* heterozygous mice. This hypothesis was tested by combining *Mef2cAHF*: *Cre* with *Gata4*^*fl/+*^. Surprisingly, OFT misalignment with DORV was only observed in 1 out of 22 embryos and none of the littermate controls (Fig 2I and 2I’ vs. 2H and 2H’, P = 1). We next tested if Gata4 is required in the pSHF for OFT alignment in in pSHF-specific *Gata4* heterozygous mice by crossing *Osr1-CreER*^*T/+*^ with *Gata4*^*fl/+*^ [44, 45]. CreER^T^ was activated by TMX administration at E7.5 and E8.5 in *Osr1-CreER*^*T/+*^; *Gata4*^*fl/+*^ embryos to results in pSHF Gata4 happloinsufficiency [44]. Similarly, neither *Gata4*^*fl*/+^*;Osr1-CreER*^*T/+*^ embryos (0/5) nor littermate control *Gata4*^*fl/+*^ embryos (0/6) demonstrated OFT misalignments at E14.5 (Fig 2J and 2J’ vs. 2H and 2H’, P = 1). However, right ventricular hypoplasia was observed in 5 out of 8 embryos (62.5%, Fig 2J and 2J’ vs. 2H and 2H’). Nonetheless, these results demonstrated that *Gata4* haploinsufficiency in either aSHF or pSHF supported normal OFT alignment.

Previous studies have shown that Hh signal-receiving progenitors localized in both the aSHF and the pSHF, and regulate the migration of SHF toward the OFT and inflow tract (IFT) to form the pulmonary artery and the atrial septum respectively [46–48]. We combined *Gli1-CreER*^*T2/+*^ with *Gata4*^*fl/+*^ to create *Gata4* haploinsufficiency in SHF Hh signal-receiving progenitors. CreER^T2^ was activated by TMX administration at E7.5 and E8.5 in *Gli1-CreER*^*T2/+*^*; Gata4*^*fl/+*^ embryos. The reduced expression of *Gata4* by the deletion of *Gli1-CreER*^*T2*^ recombination was confirmed by realtime-PCR using the SHF tissue of E9.5 embryos (Fig 1S–1G). With TMX administration at E7.5 and E8.5, 66.7% of *Gli1-CreER*^*T2/+*^*; Gata4*^*fl/+*^ embryos displayed DORV, while the littermate control *Gata4*^*fl/+*^ embryos displayed normal OFT alignment (Fig 2K and 2K’ vs. 2H, 2H’, 8/12 vs. 0/15, P = 0.0002). Blue ink was injected in the pulmonary artery of the *Gata4*^*fl/+*^ E14.5 embryos, resulting in the staining of the pulmonary artery and the right ventricle. However, the *Gli1-CreER*^*T2/+*^*; Gata4*^*fl/+*^ embryos showed staining in not only the right ventricle and the pulmonary artery, but also the aortic artery, which confirms the phenotype of DORV in these embryos (Fig 2L and 2M). In addition, when the embryos were given TMX at E8.5 and E9.5, normal OFT alignment was observed in all *Gli1-CreER*^*T2/+*^*; Gata4*^*fl/+*^ embryos (Table 1). To exclude the possibility that the phenotype might be due to the double heterozygosity for *Gata4* and *Gli1*, the phenotype of the *Gli1-CreER*^*T2/+*^*; Gata4*^*fl/+*^ embryos without TMX treatment was examined. There were no heart defects observed in the embryos at E14.5 (Table 1). Considering that TMX activates expression 12 h after injection and that the action lasts for 36 hours [49, 50], we concluded that *Gata4* is required in the SHF Hedgehog (Hh) signal-receiving progenitors from E8 to E10.5 for proper OFT alignment.

**Table 1:** Incidence of OFT defect in Gata4 mutant embryos.

Genotype	OFT defect	Total	Type	vs. control	p value
Conditional Gata4 mutant embryos					
*Gata4*^*+/-*^*;EIIa-Cre*^*+/-*^	11	18	DORV, OA	*Gata4*^*fl/+*^ (0/13)	0.0004
*Gata4*^*fl/+*^*;Tnt-Cre*^*+/-*^	0	12	——	*Gata4*^*fl/+*^ (0/9)	1
*Gata4*^*fl/+*^*; Mef2cAHF*::*Cre*	1	22	——	*Gata4*^*fl/+*^ (0/15)	1
^1^*Gata4*^*fl/+*^*; Wnt1-CreER*^*T/+*^	0	24	——	*Gata4*^*fl/+*^ (0/16)	1
^1^*Gata4*^*fl/+*^*;Osr1*^*GCE/+*^	0	5	——	*Gata4*^*fl/+*^ (0/6)	1
*Gata4*^*fl/+*^*;Nfatc1-Cre*^*+/-*^	1	15	DORV	*Gata4*^*fl/+*^ (0/10)	1
^1^*Gata4*^*fl/+*^*;Gli1-CreER*^*T2/+*^	8	12	DORV, OA	*Gata4*^*fl/+*^ (0/15)	0.0002
^2^*Gata4*^*fl/+*^*;Gli1-CreER*^*T2/+*^	0	9	——	*Gata4*^*fl/+*^ (0/9)	1
^3^*Gata4*^*fl/+*^*;Gli1-CreER*^*T2/+*^	0	7	——	*Gata4*^*fl/+*^ (0/9)	1
^1^*Pten*^*fl/+*^*;Gli1-CreER*^*T2/+*^	1	20	DORV	*Pten*^*fl/+*^ (0/7)	1
^1^*SmoM2*^*fl/+*^*;Gli1-CreER*^*T2/+*^	2	7	DORV	*SmoM2*^*fl/+*^ (0/7)	0.5677
*Pten—Gata4* compound mutant embryos					
^1^*Gata4*^*fl/+*^*;Pten*^*fl/+*^*; Gli1-CreER*^*T2/+*^	6	20	DORV	*Pten*^*fl/+*^*;Gli1-CreER*^*T2/+*^(1/20) *Gata4*^*fl/+*^*;Gli1-CreER*^*T2/+*^(12/29)	0.0915 0.5495
*Smo—Gata4* compound mutant embryos					
^1^*Gata4*^*fl/+*^*;Smo*^*fl/+*^*; Gli1-CreER*^*T2/+*^	5	9	DORV, OA, PTA	*Smo*^*fl/+*^*;Gli1*^*cre/+*^ (0/7) *Gata4*^*fl/+*^*;Gli1-CreER*^*T2/+*^(4/6)	0.0337 1
^1^*Gata4*^*fl/+*^*;SmoM2*^*fl/+*^*;Gli1-CreER*^*T2/+*^	0	9	——	*SmoM2*^*fl/+*^*;Gli1-CreER*^*T2/+*^(2/7) *Gata4*^*fl/+*^*;Gli1-CreER*^*T2/+*^(7/12)	0.1750 0.0071
*Gata4*^*+/-*^*;Smo*^*+/-*^	5	7	DORV, OA	*Gata4*^*+/-*^ (1/5) *Smo*^*+/-*^ (0/4)	0.2424 0.0606

NOTE

^1^ TMX E7.5+8.5

^2^ TMX E8.5+9.5

^3^ No treatment

### Rescue of SHF proliferation by disruption of *Pten* does not rescue DORV in *Gata4* mutant embryos

**Fig 3:**
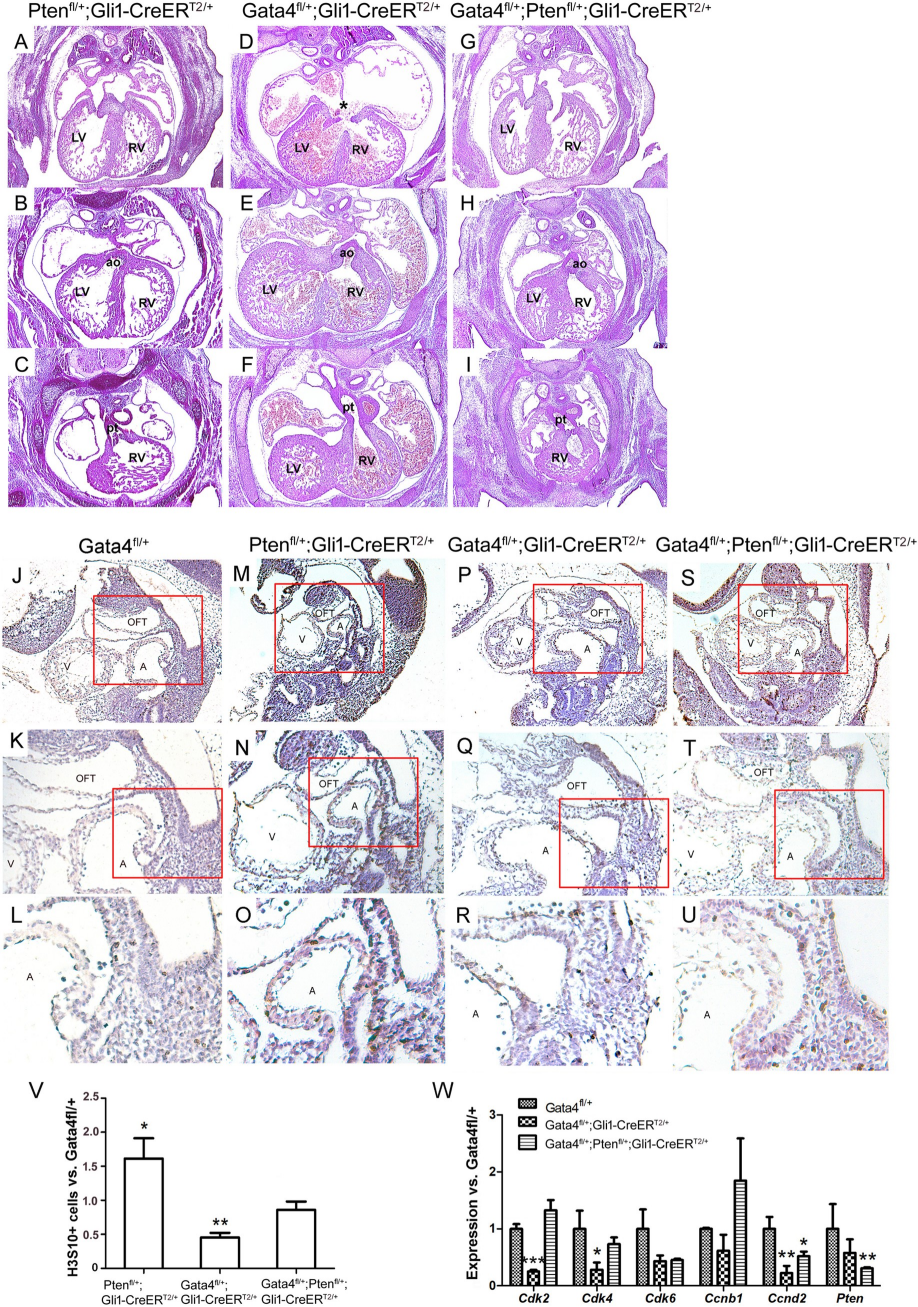
Genetically targeted ablation of Pten rescues atrioventricular septal defect. (A-I) Histology of Gata4 transgenic mouse embryonic hearts at E14.5. Two panel images of each embryonic heart were used to show the DORV phenotypes. The panels B-H showed that the opening of AO connected to the right ventricle in the Gata4 haploinsufficency (E and H), while in control embryos the AO opened to the left ventricle (B). The lower panel C-I showed the opening of PT connecting to the right ventricle. LV, left ventricle; RV, right ventricle; ao, aorta artery; pt, pulmonary trunk. Magnification: 40X. (J-U) H3S10 expression was detected in Gata4 transgenic mouse embryos by IHC at E9.5. Red rectangle area was amplified below. Magnification for panels J, M, P and S is 100X. Magnification for panels K, N, Q and T is 200X. Magnification for panels L, O, R and U is 400X. (V) Quantification of H3S10 labelled cells. Data is expressed as ratio to *Gata4*^*fl/+*^. Data is presented as Mean±SEM, **p<0.05, ***p<0.01, n = 4, compared with the *Gata4*^*fl/+*^ embryos. (W) Gene expression in the aSHF of *Gata4*^*fl/+*^, *Gata4*^*fl/+*^*;Gli1-CreER*^*T2/+*^,or *Gata4*^*fl/+*^*;Pten*^*fl/+*^*;Gli1-CreER*^*T2/+*^ embryos was measured by real-time PCR. Data is presented as Mean±SEM, n = 4, *P < 0.1, **P < 0.05 and ***P < 0.01 compared with the expression in *Gata4*^*fl/+*^ embryos.

Our previous study demonstrated that *Gata4* mutants disrupted cell cycle progression in the pSHF cardiac precursors resulting in atrial septal defects and genetically targeted downregulation of *Pten* rescued the proliferation defects in SHF of the *Gata4* heterozygotes [35]. Would the defected cell cycle by *Gata4* mutants lead to OFT alignment defects? In order to answer this question, the analysis was conducted to qualify if *Pten* downregulation (TMX at E7.5 and E8.5), could also rescue DORV in Hh-receiving cell-specific *Gata4* heterozygotes. Decreased dosage of *Pten* caused DORV in only 1 of the 20 embryos, and none with ASD (Fig 3G, 3H and 3I). Consistent with the previous report, ASD in *Gli1-CreER*^*T2/+*^*; Gata4*^*fl/+*^ embryos was rescued by *Pten* downregulation (Fig 3G vs. 3D, 1/20 in *Gli1-CreER*^*T2/+*^*; Gata4*^*fl/+*^*;Pten*^*fl/+*^ vs. 14/29 in *Gli1*^*Cre-ERT2/+*^*;Gata4*^*fl/+*^, P = 0.0013), but the *Gli1-CreER*^*T2/+*^*; Gata4*^*fl/+*^*;Pten*^*fl/+*^ embryos displayed DORV, consistent with the incidence rate from *Gli1-CreER*^*T2/+*^*; Gata4*^*fl/+*^ embryos (Fig 3H vs. 3E, 12/29 vs. 6/20, Table 1, P = 0.5495). We next performed immunohistochemical (IHC) staining for H3S10 phosphorylation to assess the cell proliferation in the SHF at E10.5. This showed a significantly less percentile of H3S10+ cells in the SHF of the *Gli1-CreER*^*T2/+*^*; Gata4*^*fl/+*^ embryos versus the *Gata4*^*fl/+*^ (Fig 3R vs. 3L and Fig 3V, P = 0.013), suggesting a proliferation defect. However, this proliferation defect was restored in the *Gli1-CreER*^*T2/+*^*; Gata4*^*fl/+*^*; Pten*^*fl/+*^ embryos (Fig 3U vs. 3L and Fig 3V, P = 0.500 vs. *Gata4*^*fl/+*^; P = 0.062 vs. *Gli1-CreER*^*T2/+*^*; Pten*^*fl/+*^). Consistently, expression of the cell proliferation genes including *Cdk2*, *Cdk4* and *Ccnd2* was lower in the *Gli1-CreER*^*T2/+*^*; Gata4*^*fl/+*^ embryos but was restored to normal levels with a *Pten* knockdown (Fig 3W). This data suggested that correction of the SHF proliferation defects failed to rescue the OFT misalignment of the *Gata4* mutant embryos, and thus different mechanisms were involved in the regulations of *Gata4* for atrial septal and OFT.

### Gata4 acts upstream of Hh signaling in OFT development

**Fig 4:**
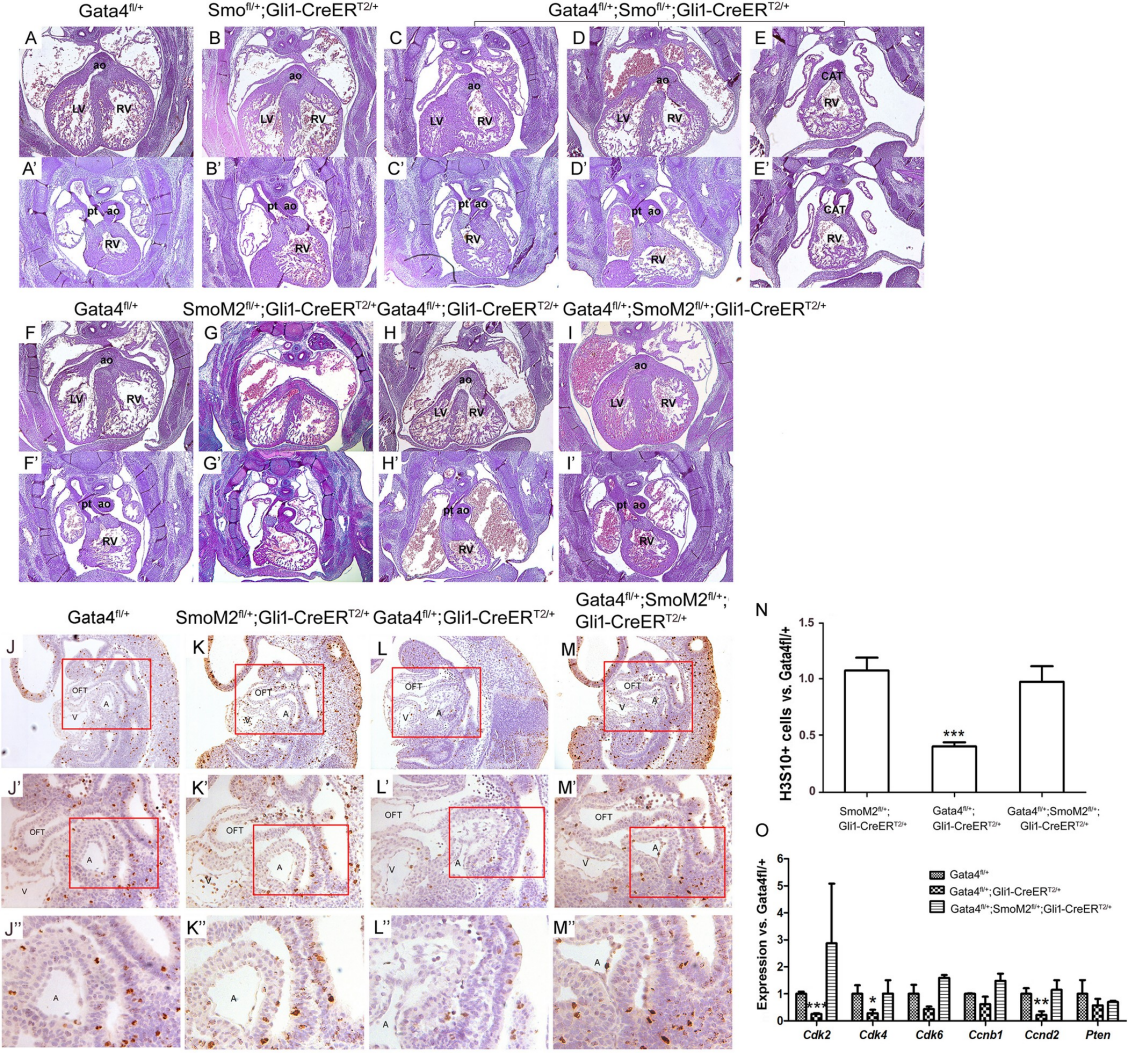
Gata4 acts upstream of Hh signaling pathway. (A-I’) Histology of Gata4 transgenic mouse embryo heart at E14.5. Two panel images of each embryonic heart were used to show the DORV phenotypes. The upper panels showed that the opening of AO connected to the right ventricle (C, DORV), or override on the ventricular septum (D and H, OA), or shared a common trunk with the PT which opens to the right ventricle (E, CAT or PTA) in the Gata4 haploinsufficency, while in control embryos the AO opened to the left ventricle (A, B, F, G and I). The lower panel showed the opening of PT connecting to the right ventricle (A’-I’). LV, left ventricle; RV, right ventricle; ao, aorta artery, pt, pulmonary trunk; CAT, common artery trunk. Magnification: 40X. (J-M’) H3S10 expression was detected in Gata4 transgenic mouse embryos by IHC at E9.5. Red rectangle area was amplified below. (J-M) Magnification: 100X. (J’-M’) Magnification: 200X. (J”-M”) Magnification: 400X. (N) Quantification of H3S10 labelled cells. Data is expressed as ratio to *Gata4*^*fl/+*^. Data is presented as Mean±SEM, ***p<0.01, n = 3, compared with the *Gata4*^*fl/+*^ embryos. (O) Gene expression in the aSHF of *Gata4*^*fl/+*^, *Gata4*^*fl/+*^*;Gli1-CreER*^*T2/+*^ or *Gata4*^*fl/+*^*;SmoM2*^*fl/+*^*;Gli1-CreER*^*T2/+*^ embryos was measured by real-time PCR. Data is presented as Mean±SEM, n = 3, *P < 0.1, **P < 0.05, ***P < 0.01, compared with the expression in *Gata4*^*fl/+*^ embryos.

We have previously reported that *Gata4* acts upstream of Hh-signaling for atrial septation [35]. The requirement of *Gata4* in *Hh-*receiving cells for OFT alignment suggested that *Gata4* and *Hh* signaling might interact genetically in the SHF for OFT development. This hypothesis was tested in the *Gata4* and *Smo* compound heterozygotes (*Gli1-CreER*^*T2/+*^*; Gata4*^*fl/+*^*; Smo*^*fl/+*^) versus the littermate controls (*Gli1-CreER*^*T2/+*^*; Gata4*^*fl/+*^; or *Gli1-CreER*^*T2/+*^*; Smo*^*fl/+*^) both induced by TMX administration at E7.5 and E8.5. Consistent OFT defects were observed in compound *Gata4; Smo* haploinsufficient embryos (*Gli1-CreER*^*T2/+*^*; Gata4*^*fl/+*^*; Smo*^*fl/+*^) (5/9, Fig 4C–4E) whereas no OFT defects were observed in *Gli1-CreER*^*T2/+*^*; Smo*^*fl/+*^ embryos (0/7, Fig 4B and 4B’; P = 0.0337). The total incidence of OFT defects occurred in the *Gli1-CreER*^*T2/+*^*; Gata4*^*fl/+*^*; Smo*^*fl/+*^was not different from in the *Gli1-CreER*^*T2/+*^*; Gata4*^*fl/+*^ embryos (Fig 4C–4E, 5/9 vs. 4/6, P = 0.7326). However, a larger range of OFT defects was observed in *Gli1-CreER*^*T2/+*^*; Gata4*^*fl/+*^*; Smo*^*fl/+*^ embryos, including DORV (3 out of 5, Fig 4C and 4C’), OA (1 out of 5, Fig 4D and 4D’) and persistent truncus arteriosus (PTA) (1 out of 5, Fig 4E and 4E’). PTA, caused by a combined defect of alignment and separation, was only observed in *Gli1-CreER*^*T2/+*^*; Gata4*^*fl/+*^*; Smo*^*fl/+*^. This result suggested an interaction between *Gata4* and Hh-signaling in OFT development.

We tested the hypothesis that *Gata4* acts upstream of Hh-signaling for OFT development using a genetic epistasis study. The purpose was to understand if increased Hh-signaling via a constitutively activated Smo mutant, *SmoM2* [35], induced by TMX administration at E7.5 and E8.5, could rescue the OFT misalignment in *Gata4*-heterozygotes. DORV was observed in 28.6% of littermate control *Gli1-CreER*^*T2/+*^*; SmoM2*^*fl/+*^embryos (2/7) (Fig 4G and 4G’) and 58.3% of littermate control *Gli1-CreER*^*T2/+*^*; Gata4*^*fl/+*^ embryos at E14.5 (7/12) (Fig 4H and 4H’). In contrast, none of *Gli1-CreER*^*T2/+*^*; Gata4*^*fl/+*^*; SmoM2*^*fl/+*^ embryos showed DORV (Fig 4I and 4I’), indicating significant rescue by *Gli1-CreER*^*T2/+*^*; SmoM2*^*fl/+*^(Fig 4I vs Fig 4H, P = 0.0071, Table 1). This results demonstrated rescue of DORV in *Gata4*-mutant embryos by constitutive Hh signaling.

Next, IHC staining for H3S10 phosphorylation in the SHF was performed to determine if the cell proliferation defects observed in the *Gli1-CreER*^*T2/+*^*; Gata4*^*fl/+*^ were rescued by overactivation of Hh-signaling. Clearly, the cell proliferation defects, indicated by less percentile of H3S10+ cells, observed in the SHF of the *Gli1-CreER*^*T2/+*^*; Gata4*^*fl/+*^ (Fig 4L” vs. 4J” and Fig 4N, P<0.01 vs. *Gata4*^*fl/+*^) were recovered by *Gli1-CreER*^*T2/+*^*; SmoM2*^*fl/+*^ (Fig 4M” vs. 4J” and Fig 4N, P>0.05 vs. *Gata4*^*fl/+*^). Consistently, gene expression was downregulated for multiple cell proliferation genes including the *Cdk2*, Cdk4, *Cdk6* and *Ccnd*2 in the SHF of *Gli1-CreER*^*T2/+*^*; Gata4*^*fl/+*^ comparing to the *Gata4*^*fl/+*^ embryos, which was recovered in the *Gli1-CreER*^*T2/+*^*; Gata4*^*fl/+*^*; SmoM2*^*fl/+*^ embryos (Fig 4O). These results suggested that overactivating the Hh-signaling rescued proliferation defects in the SHF of Gata4 haploinsufficiency.

### *Gata4* is required for the contribution of Hh-receiving cells to the OFT

**Fig 5:**
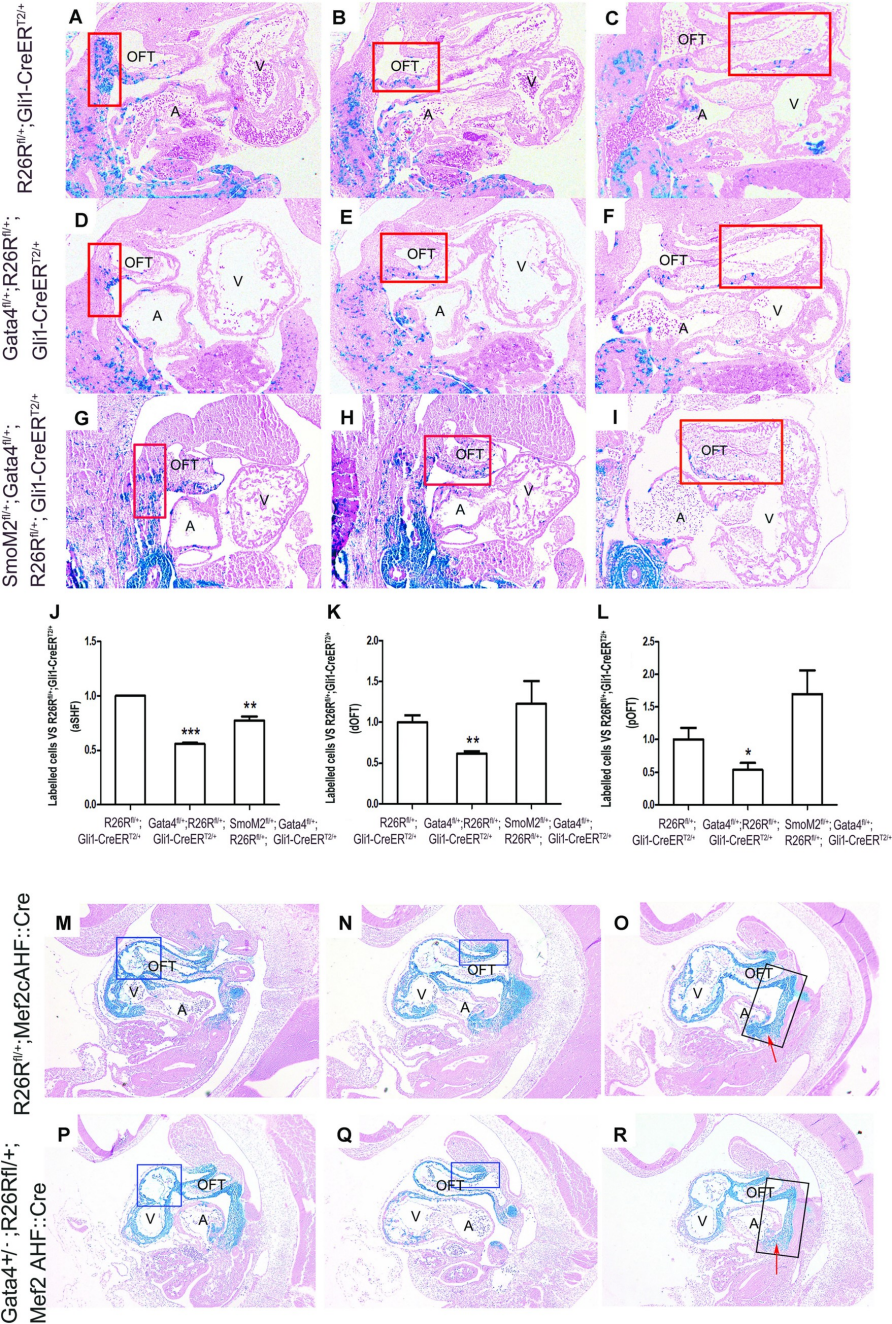
*Gata4* is required for the contribution of Hh-receiving cells to the OFT. The embryos were given TMX at E7.5 and E8.5 and the β-galactosidase expression was evaluated at E11.5. (A-I) LacZ staining of Gli1-expressing cells in Gata4 transgenic mouse embryos at E11.5 focusing on aSHF (A, D and G), dOFT (B, E and H) and pOFT (C,F and I). Magnification: 100X (J-L) Quantification of stained cells within selected regions. Data is expressed as ratio to *R26R*^*fl/+*^*; Gli1-CreER*^*T2/+*^. Data is presented as Mean±SE, *p<0.1, ** p<0.05 and *** p<0.01 vs. *Gata4*^*fl/+*^, n = 3–5, One-way ANOVA. (M-R) LacZ staining of cells with Mef2cAHF::Cre expression in Gata4 transgenic mouse embryos at E10.5. The red arrow indicated a developing DMP region. Magnification: 100X.

Hh signaling has been reported to regulate the migration of SHF Hh-receiving cells toward the arterial pole of the heart [46]. We therefore hypothesized that *Gata4* drives SHF Hh-receiving cells migration toward the developing OFT. This hypothesis was tested by using genetic inducible fate mapping (GIFM) [51]. The Hh-receiving lineage cells were marked by TMX administration at E7.5 and E8.5 (*Gli1-CreER*^*T2/+*^*; R26R*^*fl/+)*^ and *β-gal* expression was evaluated at E11.5. We assessed if there was less migrating Hh-receiving SHF cells migrating through the distal OFT (dOFT) towards the proximal OFT (pOFT) in the Gata4 haploinsufficient embryos (*Gli1-CreER*^*T2/+*^*; Gata4*^*fl/+*^*; R26R*^*fl/+*^) than the control embryos (*Gli1-CreER*^*T2/+*^*; R26R*^*fl/+*^), and if this defects rescued in *Gli1-CreER*^*T2/+*^*; R26R*^*fl/+*^*; Gata4*^*fl/+*^*; SmoM2*^*fl/+*^ embryos. Previous reports indicate a decreased number of Hh-receiving cells in the pSHF at E9.5 associated with developing defects of DMP in the *Gata4*^*fl/+*^*; R26R*^*fl/+*^*; Gli1*^*Cre-ERT2/+*^embryos [35]. In concurrence, significantly less Hh-receiving cells within the aSHF region (Fig 5A vs. 5D and Fig 5J, 334.0 ± 1.4 vs. 186.7 ± 4.9, P = 0.001) of the *Gli1-CreER*^*T2/+*^*; Gata4*^*fl/+*^*; R26R*^*fl/+*^ embryos was also observed. The cells of Hh-receiving lineage were analyzed in the developing OFT at this stage. By counting the number of *β-galactosidase*-expressing cells in the proximal half and the distal half of the OFT myocardium of the *Gata4*^*fl/+*^*; R26R*^*fl/+*^ embryos, both of the regions of the *Gli1-CreER*^*T2/+*^*; Gata4*^*fl/+*^*; R26R*^*fl/+*^ had less *β-galactosidase*-expressing cells than the littermate controls (Fig 5B vs. 5E and 5K, 91.7 ± 9.2 vs. 56.7 ± 1.4, P = 0.013 for dOFT; Fig 5C vs. 5F and 5L, 49.7 ± 10.6 vs. 26.7 ± 6.4, P = 0.091 for pOFT). Importantly, the lower number of Hh-receiving cells in the *Gli1-CreER*^*T2/+*^*; Gata4*^*fl/+*^ was partially recovered by overactivating the Hh-signaling in the aSHF (Fig 5A vs. 5G and 5J, *Gli1-CreER*^*T2/+*^*; R26R*^*fl/+*^ vs. *Gli1-CreER*^*T2/+*^*; R26R*^*fl/+*^*; Gata4*^*fl/+*^*; SmoM2*^*fl/+*^: 334.0 ± 1.4 vs. 258 ± 18.4, P = 0.028; Fig 5G vs. 5D and 5J, *Gli1-CreER*^*T2/+*^*; R26R*^*fl/+*^*; Gata4*^*fl/+*^*; SmoM2*^*fl/+*^ vs. *Gli1-CreER*^*T2/+*^*; R26R*^*fl/+*^: 258 ± 18.4 vs. 186.7 ± 4.9, P = 0.034). There was also complete restoration of the amount of *β-galactosidase*-expressing cells in the dOFT (Fig 5B vs. 5H and 5K, *Gli1-CreER*^*T2/+*^*; R26R*^*fl/+*^ vs. *Gli1-CreER*^*T2/+*^*; R26R*^*fl/+*^*; Gata4*^*fl/+*^*; SmoM2*^*fl/+*^: 91.7 ± 9.2 vs. 109.3 ± 19.8, P = 0.505) and the pOFT (Fig 5C vs. 5I and 5L, *Gli1-CreER*^*T2/+*^*; R26R*^*fl/+*^ vs. *Gli1-CreER*^*T2/+*^*; R26R*^*fl/+*^*; Gata4*^*fl/+*^*; SmoM2*^*fl/+*^: 49.7 ± 10.6 vs. 84.3 ± 15.7, P = 0.161).

To examine if a Gata4 heterozygous influenced the SHF cell recruitment within the proximal OFT, we analyzed the fate map of SHF lineage cells in the OFT of the *Gata4* heterozygotes. Defined by Mef2cAHF::Cre driven β*-galactosidase*-expressing cells, the total number of the SHF lineage cells within the proximal and distal half of the OFT were compared between the *Mef2cAHF*::*Cre; R26R*^*fl*/+^*;Gata4*^*+/-*^; and the *Mef2cAHF*::*Cre;R24R*^*fl/+*^ embryos at E10.5. The number of SHF lineage cells populating the pOFT of the *Mef2cAHF*::*Cre;Gata4*^*+/-*^*; R26R*^*fl/+*^ embryos was significantly less than those in the control *Mef2cAHF*::*Cre; R26R*^*fl/+*^ embryos (Fig 5M vs. 5P); however, this decrement was not observed in the distal OFT (Fig 5N vs. 5Q). The distribution pattern of the SHF lineage was not different in the *Mef2cAHF*::*Cre;Gata4*^*+/-*^*; R26R*^*fl/+*^ and the *Mef2cAHF*::*Cre;R26R*^*fl/+*^embryos (Fig 5O vs. 5R). Fewer cells were observed to populate the developing dorsal mesocardium protrusion (DMP) in *Mef2cAHF*::*Cre; Gata4*^*+/-*^*; R26R*^*fl*/+^*(*red arrow, Fig 5O vs. 5R). This was consistent with the previous report that Gata4 is required in the SHF for the DMP [35]. These results demonstrated the requirement of Gata4 for the SHF lineage cells populating in the developing OFT.

### Gata4 is involved in the endothelial-to-mesenchymal transformation (EMT) and mesenchymal cell proliferation for OFT cushion development

**Fig 6:**
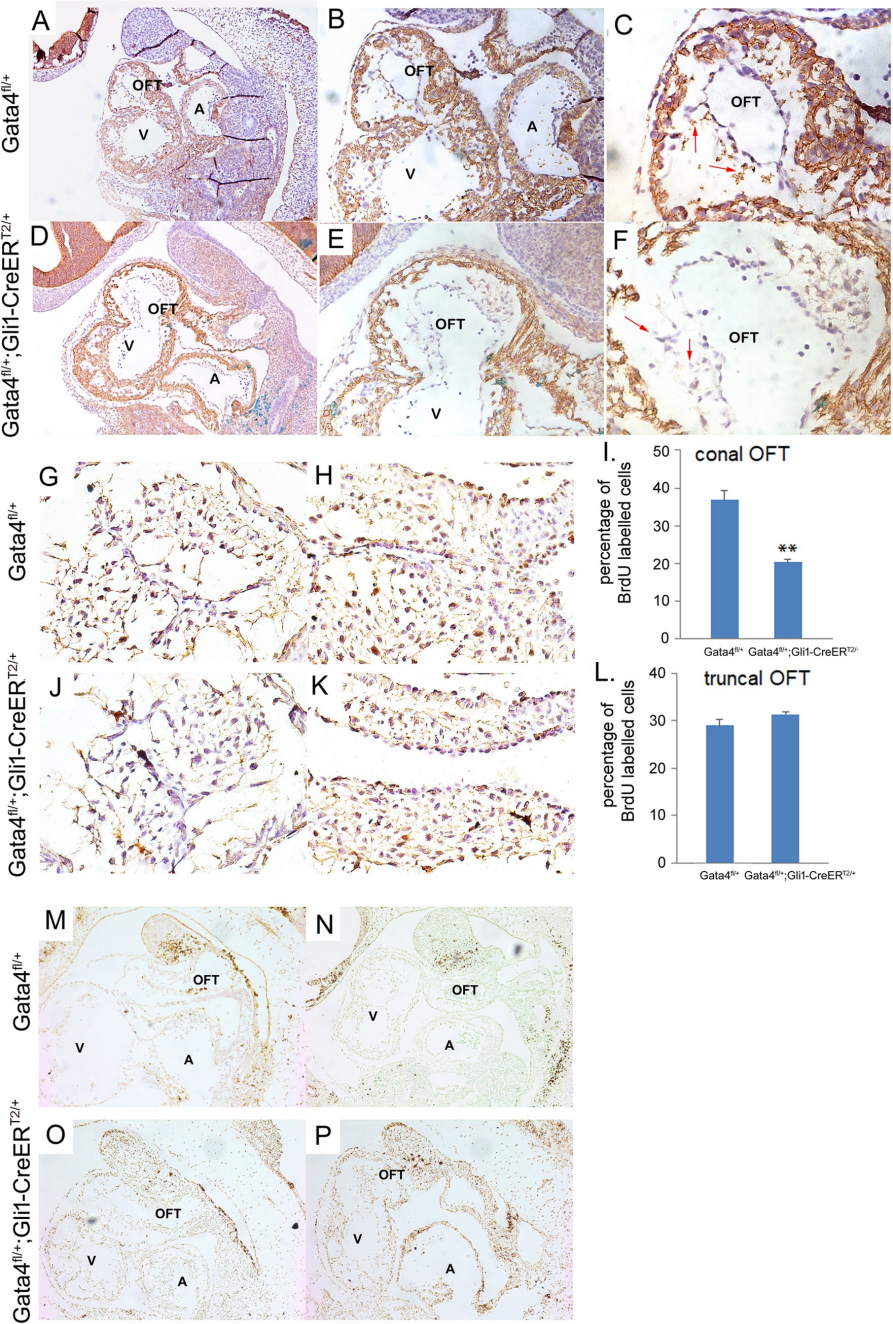
Gata4 regulates cell proliferation in conal OFT. (A-F) IHC staining for N-Cadherin in *R26R*^*fl/+*^*; Gata4*^*fl/+*^ and *R26R*^*fl/+*^*; Gata4*^*fl/+*^*; Gli1-CreER*^*T2/+*^ embryos at E9.5. LacZ staining was performed before the IHC staining, which indicated the active Cre recombination for specifically Gata4 knocking-down. The Hh-receving cells were shown to populate at the conal OFT as early as E14.5. Magnification: A and D, 100X; B and E, 200X, C and F, 400X. (G-L) BrdU staining in conal OFT and truncal OFT in *Gata4*^*fl/+*^*; Gli1-CreER*^*T2/+*^ embryos and control embryos at E11.5. Magnification: 400X. (I and L) Quantification of BrdU labelled cells. Data is expressed as percentile of *Gata4*^*fl/+*^. Read arrow indicate the cells undergoing EMT. Data is presented as Mean±SE, **p<0.05, n = 3–5, compared with the *Gata4*^*fl/+*^ embryos. (M-P) TUNEL staining in both *Gata4*^*fl/+*^*; Gli1*^*Cre-ERT2/+*^ embryos and control embryos at E11.5. OFT: out flow tract; A: atrium; V: ventricle. Magnification: 100X.

The role of Gata4 in the EMT for the endocardial cushion development has been well described previously [20, 23, 52]. Since less SHF lineage cells populating in the developing OFT myocardium were observed, we asked if this defect would affect the sequential events such as EMT, cell proliferation or cell survival in the developing OFT via a non-cell autonomous manner. Expression of mesenchymal marker N-cadherin was used to label the cushion cells undergoing EMT. LacZ staining was performed before the IHC staining, which indicated the active Cre recombination for specifically Gata4 knocking-down. The Hh-receiving cells were shown to populate at the conal OFT as early as E14.5. Significantly less N-cadherin staining of the conal OFT cushion cells in the *Gli1-CreER*^*T2/+*^*; Gata4*^*fl/+*^*; R26R*^*fl/+*^ embryos versus the *Gata4*^*fl/+*^*; R26R*^*fl/+*^ littermate control embryos at E10.5 (Fig 6A–6C vs. 6D–6E) was observed, suggesting that the EMT process of the OFT cushion was inhibited by the lower Gata4 expression in Hh-receiving cells. Cell proliferation was examined by BrdU incorporation at E11.5. *Gli1-CreER*^*T2/+*^*; Gata4*^*fl/+*^ embryos demonstrated 17% fewer BrdU-positive SHF cells in the OFT conal cushion (Fig 6G vs. 6J and 6I; *P* = 0.0134), but not the OFT truncal cushion (Fig 6H vs. 6K and 6L; *P* = 0.1998), when compared to the littermate *Gata4*^*fl/+*^embryos at E11.5. Cell death were assessed by TUNEL staining and no differences in either the conal or truncal cushion between *Gli1-CreER*^*T2/+*^*; Gata4*^*fl/+*^ and the *Gata4*^*fl/+*^embryos was observed (Fig 6M–6P). Together, these results demonstrated that Gata4 is required for normal cell EMT and proliferation in OFT conal cushion development, possibly through a non-cell autonomous manner.

### Gata6 was overexpressed in the SHF of the Gata4 transgenic mouse embryos

**Fig 7:**
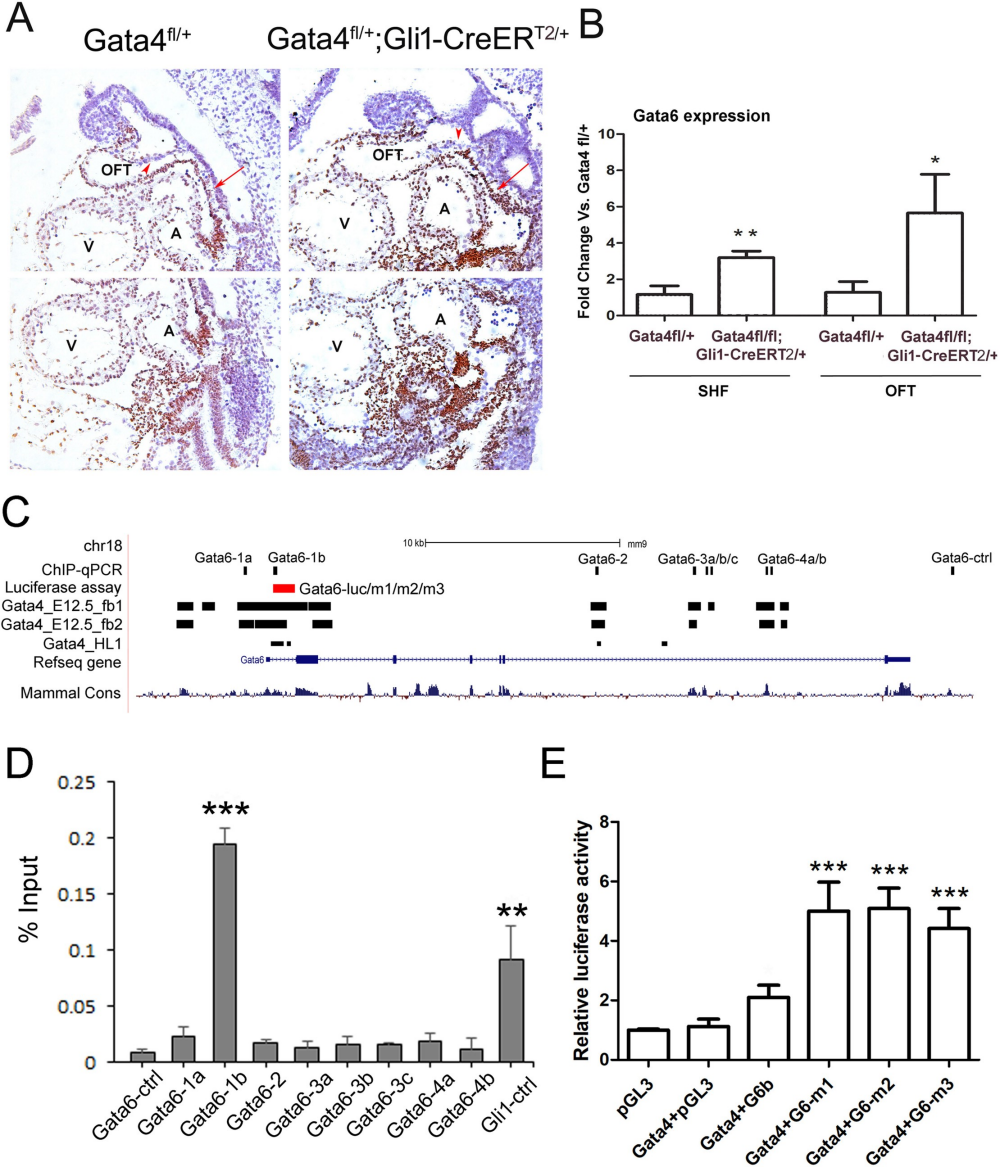
Gata6 is a repressing target of Gata4 in the SHF. (A) IHC of the Gata6 in *Gata4*^*fl/+*^ and *Gata4*^*fl/+*^*; Gli1-CreER*^*T2/+*^ embryos at E9.5. The arrowhead indicated the NCCs-derived cells and the arrow indicates the splanchnic mesoderm. Magnification: 200X. (B) Gata6 was measured by realtime-PCR in the micro-dissected SHF and the OFT of the *Gata4*^*fl/+*^ and *Gata4*^*fl/fl*^*; Gli1-CreER*^*T2/+*^ embryos at E9.5. *p<0.1 vs. *Gata4*^*fl/+*^, **p<0.05, n = 3–5, compared with the expression in *Gata4*^*fl/+*^ embryos. OFT: out flow tract; A: atrium; V: ventricle. (C) Schematic of the mouse Gata6 genomic locus including Gata4-binding regions and the cloned genomic fragments used for Gata4 regulation assays (luciferase reporter assay and ChIP-qPCR). (D) Enrichment of Gata4-responsive Gata6 genomic fragments in the SHF by Gata4 ChIP-qPCR. Results are presented as mean ± SEM; n = 4; **P < 0.05 vs. Gata6-ctrl, ***P < 0.01, compared with Gata6-ctrl. (E) Gata4-stimulated firefly luciferase activity in wild-type Gata6 and Gata6 mutant fragments. Results are presented as mean ± SEM; n = 4; ***P < 0.01, compared with Gata4+pGL3.

Because *Gata4* and *Gata6* double mutant embryos display PTA [33], *Gata6* expression in *Gata4* mutants was examined. Gata6 was expressed in the heart, the OFT and strongly in the splanchnic mesoderm (Fig 7A, arrow), but not neural crest cell derivatives (Fig 7A, arrowhead) of the *Gata4*^*fl/+*^ embryo at E9.5. In *Gata4* knockdown embryos specifically in the Hh-receiving cells, the *Gata6* expression domain was strongly enhanced in the OFT and the splanchnic mesoderm. Consistently, enhanced expression of *Gata6* in the OFT and the SHF of the *Gata4*^*fl/fl*^*; Gli1-CreER*^*T2/+*^ was further confirmed by the real-time PCR at the mRNA level (Fig 7B). The *Gata6* expression in the SHF of *Gata4*^*fl/fl*^*; Gli1-CreER*^*T2/+*^ mouse embryo was increased by 1.7-fold comparing to the control *Gata4*^*fl/+*^ embryos (P<0.05). *Gata6* expression in the OFT of the *Gata4*^*fl/fl*^*; Gli1-CreER*^*T2/+*^ mouse embryo was increased by 3.4-fold comparing to the littermate control (P<0.01). These results suggested a negative association between the expression of *Gata4* and *Gata6* in the SHF and developing OFT.

We tested the possibility of Gata4 regulating the expression of Gata6 in the SHF as a repressor by ChIP-qPCR using the microdissected SHF from the E9.5 wildtype mouse embryos. The *Gata6* loci was bioinformatically interrogated for potential Gata4-responsive elements using the overlap of evolutionary conservation and Gata4 occupancy in HL-1 cells or embryonic mouse hearts [53, 54] (Fig 7C). Eight potential Gata4-binding regions for Gata6 were screened and our previously identified Gata4 responsive Gli1 loci [35] was used as the positive control (Gli1-ctrl). The results showed enrichments of the region Gli1-ctrl and the region Gata6-1b, but not the others (Fig 7D). This suggested that the region Gata6-1b, within 1 Kbp upstream of Gata6 start codon, was responsive to Gata4 binding. Luciferase reporter assay showed no changes of firefly luciferase activity under Gata6-1b expression in HEK293 cells (Fig 7E, Gata4+G6-luc vs. Gata4+pGL3, P>0.05). However, three mutant constructs for Gata6-1b, each ablating one Gata4-binding site, significantly enhanced the luciferase activity (Fig 7E, P<0.001 for all three mutants versus the Gata4+pGL3). Together, these results place Gata4 upstream of *Gata6* as a repressor in the SHF.

## Discussion

The requirement of Gata4 for OFT development has been reported in mice and humans, and Gata4 mutations causing DORV have been reported in mice [19, 20, 33]. Here Gata4 is demonstrated required in the SHF Hh-receiving cells for OFT alignment. Our previous study has demonstrated that Gata4 is required for Hh signaling in the SHF for cell proliferation. However, the current study suggested that the cell proliferation defects in the SHF caused by Gata4 mutation may not be the only mechanism underlying the OFT misalignment. Another important contributing factor is the migration defect of the SHF cells, which were associated with disrupted Hh-signaling, as proved by the rescue of over-activating Hh-signaling. As subsequent events, Gata4 haploinsufficency in the Hh-receiving cells disrupted EMT process and cell proliferation in the conal OFT cushion, suggesting that both the cell-autonomous and non-cell autonomous effects of Gata4 drive OFT development. In addition, we demonstrated Gata4 as a repressor of *Gata6* in the SHF by identifying Gata4-responsive binding sites in its promoter regions. This result provides a molecular explanation for the severity of OFT defects observed in *Gata4* and *Gata6* double mutant embryos. These data suggested that breaking down the threshold of GATA including *Gata4* and *Gata6*, and Hh signaling tone might be associated with the severity of OFT defects.

The SHF was initially described as a progenitor field for the cardiac OFT and a rich literature has established the requirement of aSHF contributions for OFT development [5, 12, 35, 55–65]. More recently, the contribution of pSHF cardiac progenitors to the OFT and the future subpulmonary myocardium has been reported; however, the mechanistic requirement for this contribution is not well understood [46, 66–68]. The cell lineage in which Gata4 is required for OFT development has not been reported. Gata4 is expressed in both the aSHF and pSHF, although its expression is much stronger in the pSHF [35]. The decreased number of *Mef2C-AHF*::*Cre* positive cells in the proximal OFT cushion of E10.5 *Gata4*^−*/+*^ embryos demonstrated that Gata4 plays a role in adding the SHF progenitor cells to the developing OFT. Surprisingly, OFT defects were not observed in either aSHF-specific (Mef2c-AHF::Cre) or pSHF-specific (Osr1-CreER^T^) Gata4 haploinsufficiency. Instead, it was found that the severity of the OFT defects and incidence rate in embryos with *Gata4* haploinsufficiency in *Hh*-receiving cells were identical to those in *Gata4*^−*/+*^ embryos. Because Hh-receiving cells are located throughout the SHF, these observations suggested that Gata4 is required in both pSHF and aSHF progenitor cells for OFT alignment.

Evidence was provided that Gata4 acts upstream of Hh-signaling in the SHF for OFT development. The *Gata4*^−*/+*^ embryos have combined phenotypes of ASD and DORV [35]. This study, adding new knowledge to the previous [35], have disclosure that Gata4-Hh-signaling plays active, but different, roles at the venous and atrial pole of the developing heart. In the SHF, Gata4-Hh-signaling controls cell cycle progression and thereby the proliferation of the cardiac progenitors. At the venous pole, diminished Gata4-Hh signaling for cell cycle regulation is balanced by Pten through transcriptional inhibition of Cyclin D4 and Cdk4 [13, 35], as DMP hypoplasia and SHF cell cycle defects are rescued by *Pten* knockdown [13, 35]. At the atrial pole, the *Pten* knockdown was able to rescue the cell cycle defects in the SHF, but failed to rescue DORV or OA defects in *Gata4* heterozygous mutants. This observation suggests that correction of SHF cell proliferation is not sufficient to support a normal OFT development in Gata4 mutants. In this study, increased apoptosis was not observed in the SHF of *Gata4* heterozygote mutant embryos [35]. Indeed, cell migration under the regulation of Gata4-Hh signaling is important for OFT development. However, fate mapping of the SHF using either *Mef2c-AHF*::*Cre* or the *Gli1-CreER*^*T2*^ disclosed less SHF-derived cells in the distal OFT in Gata4 mutant embryos. Specifically, there was a decreased number of SHF Hh-receiving cells throughout the migration route from the SHF into the OFT, which traveled from the dorsal mesocardium and continued through the rostral splanchnic mesoderm, past the distal OFT and reached the proximal OFT. Hh-receiving progenitors have been found to migrate from the aSHF to populate the pulmonary trunk between E9.5 to E11.5 [46], suggesting that Hh-signaling is required for SHF cell migration. The observation that DORV in Gata4 mutant embryos can be rescued by constitutive Hh-signaling is correlated with restored cell migration and cell proliferation in the SHF. Therefore, this data suggested that the normal Gata4 regulation of both the proliferation and the migration of the SHF cardiac progenitors are required for OFT development.

During development, the ventricular outlets are aligned to the ventricles by the fusion of conal cushions with the interventricular septum [69], and improper lengthening of the conal cushion may cause rotation problems resulting in misalignment. We demonstrated that Gata4 plays a non-cell autonomous role in the EMT process to give rise to the conal cushions mesenchyme. It is still unknown what specific signals the SHF-derived OFT Gli1+ myocardial cells provide for EMT. Future studies should aim to identify the ligands secreted by Hh-receiving cells. Moreover, a smaller percentage of BrdU+ cells in the conal cushion of the OFT was found at E11.5 of the *Gata4*^*fl/+*^; *Gli1*^*Cre-ERT2/+*^ embryos, suggesting Gata4 plays a role in regulating the OFT cushion cell proliferation. Inactivating N-Cadherin in the SHF resulted in hypoplastic OFT and right ventricle, associated with decreased proliferation [70, 71]. Thus, the lower number of proliferating cells might be associated with the lower expression of N-Cadherin. Overall, cellular, molecular and genetic evidence proved that Gata4-Hh signaling is required in OFT alignment via both the cell-autonomous and non-cell autonomous manners.

Although important *Gata4* transcriptional targets in the heart have been identified [13, 18, 37], *Gata4*-dependent molecular pathways required for OFT development remain unknown. *Gli1* was previously identified as a downstream target of Gata4 in the pSHF for atrial septation [35]. In the current study, it was further demonstrated that Gata4 controls Hh-signaling though *Gli1* transcriptional regulation for cell migration and OFT alignment. In addition, Gata4 was demonstrated to be a transcriptional repressor of *Gata6* in the SHF, and the Gata4-responsive sites in the Gata6 promoter region were identified. Previously, several downstream targets of Gata4 including the *Mef2c* and *Gli1* have been recognized [13, 15, 16, 18, 36, 37], while none of them respond to the inhibitory effects. *Gata6* is the first identified repressing target of Gata4, providing direct evidence that Gata4, as a transcription factor, is not only an activator but also a repressor. Enhanced *Gata6* expression in *Gata4* mutants might illustrate a compensatory feedback loop, given that *Gata6* and *Gata4* are redundant for cardiac myocyte differentiation [72, 73]. *Gata4/Gata6* compound heterozygotes displayed persistent truncus arteriosus (PTA), a severe OFT defect caused by combined alignment and OFT septation defects [33]. This study shows that *Gata4/Smo* compound heterozygotes show a similar phenotype. *Gata4* heterozygote alone does not display PTA, which might be due to the partial recovery of GATA function from enhanced *Gata6* expression. Together with previous study [33], these data suggest a threshold of *Gata4*, *Gata6*, and *Hh* signaling and that is required for OFT development. This implies that GATA TFs may be essential for the quantitative regulation of Hh signaling, and diminished GATA function or reduced GATA and Hh signaling together may cause more severe OFT defects. Future studies will focus on the quantitative relationship between GATA tone and Hh signaling tone, as well as the Gata4 dependent gene regulatory network (GRN) [74] for OFT development.

## Materials and methods

### Mouse lines

All mouse experiments were performed in a mixed B6/129/SvEv background. *Gata4*^*fl/+*^, *Gli1-CreER*^*T2/+*^, *Mef2cAHF*::*Cre*, *Smo*^*fl/+*^ mouse lines were kind gifts from Dr. Ivan Moskowitz lab (University of Chicago, Chicago). *TnT-Cre*^*+/-*^ mouse line was from Dr. Yiping Chen lab (Tulane University, New Orleans). *Nfat1c-Cre*^*+/-*^ mouse line was from Dr. Bin Zhou lab (Albert Einstein College of Medicine, Bronx, NY). The *SmoM2*^*fl/+*^, *Osr1-CreER*^*T/+*^ and *EIIa-Cre*^*+/-*^ mouse lines were purchased from the Jackson Laboratory.

### Ethics statement

Mouse experiments were completed according to a protocol reviewed and approved by the Institutional Animal Care and Use Committee of Texas A&M University (#2015–0398), in compliance with the USA Public Health Service Policy on Humane Care and Use of Laboratory Animals.

### Tamoxifen administration and X-gal staining

TMX-induced activation of *CreER*^*T2*^ was accomplished by oral gavage with two doses of 75 mg/kg TMX at E7.5 and E8.5 [44, 46]. X-gal staining of embryos was performed as described [46]. The total number of β-gal positive cells was obtained by counting those on each individual sections and adding up all through the SHF and the OFT.

### BrdU incorporation and Immunohistochemistry Staining (IHC)

Standard procedures were used for histology and IHC. For BrdU incorporation, pregnant mice were given 100mg BrdU per kg bodyweight at 10mg/mL concentration solutions at E11.25 with two doses, 3 hours and 6 hours before sacrifice, respectively. The BrdU staining was performed using a BrdU In-Situ detection kit (EMD Millipore). For TUNNEL staining, an ApopTag plus peroxidase In-Situ apoptosis detection kit was used (EMD Millipore). IHC was performed using the following antibodies: anti-Gata4 (Abcam, #ab84593), anti-Gata6 (Abcam, #ab175349), and anti-N-cadherin (Abcam, #ab18203). After incubating with the first antibody, a VECTASTAIN ABC HRP Kit (LifeSpan BioSciences, Inc) was used for detecting the protein expression signal. For counting the ratio of proliferating cells, a total of 100 random cells within the SHF and the specific OFT regions per each section were counted using the Particle Analysis tool of ImageJ and the ratio of positively stained cells was recorded. For each sample, five equivalent serial sections were counted and the averages were taken for statistical analysis.

### Micro-dissection of pSHF and RNA extraction

To obtain the pSHF splanchnic mesoderm for use in quantitative realtime-PCR, E9.5 embryos were dissected as described before [45, 75]. The heart, aSHF, and pSHF were collected separately in RNA-later, and then stored at −20°C until genotyping was completed.

### Realtime-PCR

Total RNA was extracted from the PSHF regions of mouse embryos hearts using RNeasy Mini Kit (QIAGEN), according to the manufacturer’s instructions. Two hundred ng of total RNA was reverse transcribed using a SuperScript^TM^ III Reverse Transcriptase kit from Invitrogen. qPCR was performed using a POWER SYBER Green PCR mater mix from Applied Biosystems. Results were analyzed using the delta-delta Ct method with *GAPDH* as a normalization control [76].

### Chromatin immunoprecipitation

Chromatin Immunoprecipitation was performed as described previously [35]. The ChIP assay was performed using a Gata4 antibody (Santa Cruz, #sc-1237 X). Genomic regions with potential Gata4-binding sites and negative control sites are listed in Table 2. Primers used for evaluating the enrichment of the Gata4 pull-down fragments via realtime-PCR are listed in Table 3.

**Table 2:** Genomic regions with Gata4-binding sites assessed by luciferase reporter assay and ChIP-qPCR.

	Luciferase assay				ChIP-qPCR		
Gene name	Genomic fragment	Locus	Luciferase results	Gata4-binding sites in subcloned fragments	Genomic fragment	Locus	ChIP results
Gata6	Gata6-luc	chr18: 11052898–11054024	1.51±0.97 P = 0.0945	chr18: 11052933–11053086	Gata6-1a	chr18: 11051387–11069602	0.02±0.01 P = 0.1128
	Gata6-luc/m1		0.90±0.03 P = 0.1198	chr18: 11052933–11052964	Gata6-1b	chr18: 11052900–11053090	0.19±0.01 P = 0.0141
	Gata6-luc/m2		0.93±0.03 P = 0.3169	chr18: 11053019–11053024	Gata6-2	chr18: 11069461–11069602	0.02±0.00 P = 0.1530
	Gata6-luc/m3		0.81±0.05 P = 0.3552	chr18: 11053062–11053066	Gata6-3a	chr18: 11074459–11074606	0.01±.01 P = 0.4378
					Gata6-3b	chr18: 11075106–11075234	0.02±0.01 P = 0.3491
					Gata6-3c	chr18: 11075344–11075487	0.02±0.00 P = 0.1789
					Gata6-4a	chr18: 11078180–11078329	0.02±0.01 P = 0.1773
					Gata6-4b	chr18: 11078433–11078559	0.02±0.01 P = 0.8491
					Gata6-ctrl	chr18: 11087740–11087888	0.00±0.00
					Gli1-ctrl	chr10: 126771190–126771322	0.09±0.03

All genomic coordinates are shown in mouse genome build mm9.

**Table 3:** Primers for ChIP-qPCR.

Fragment	Forward 5’ → 3’	Reverse 5’ → 3’
Gata6-1a	ATATCACTGCTGCTGCCTGG	CACACACCCCTTAGTCGCTC
Gata6-1b	ATACTCCGGACCAGCCTCC	CAAATCGCTTAGGCTCATCGG
Gata6-2	CGAGGAACATATTTTGCCTGCC	ACACCCAGCAAAGCAGAAGTG
Gata6-3a	CACTTGCCACATGCTGCAAAC	ACATTCTCTGCTACGGTGAC
Gata6-3b	CTGCCAAAAGGGTTACCAGC	TCCGGTGACACCTGTCTTTG
Gata6-3c	CTGAGGCTAGCCAGGAACTCC	CAGGCCATGGAGTTGGTCCTC
Gata6-4a	ATGCTATGCTAAGCCCAGGC	GTGCATTGAGGGCAGAGTAGA
Gata6-4b	TCTCTGCTTTCTCCTAGGGAC	TCCATGAGTTAACATTTTCCCAC
Gata6-ctrl	CCCCTGGGAGGTAACACAGAC	CCTCAGTTTCCCTGTACCCAC
Gli1-ctrl	GAGGGATACTTAGGCGGC	GTTGCAGCAAGGCCTTTAGC

### Dual Luciferase Reporter Assay

Dual Luciferase Reporter Assay was performed as described previously [35, 44, 45]. Genomic regions with potential Gata4-binding sites tested are listed in Table 2. Primers used for site-specific mutation and subclone are listed in Table 4.

**Table 4:** Primers for luciferase reporter assay.

Fragment	Forward 5’ → 3’	Reverse 5’ → 3’
Gata6-luc	GTCACCCGGGATACTCCGGACCAGCCTC	AGTCAAGCTTGAGTGAGGAACAAGACGG
Gata6-luc/m1	CCCCCAGTGCAAAGCCCACAGCCCGAGTTTCAGCGCCAAG	CTTGGCGCTGAAACTCGGGCTGTGGGCTTTGCACTGGGGG
Gata6-luc/m2	GAGAAACTTCTTTCTTGTGCCTGGGTCTGTGTGTGG	CCACACACAGACCCAGGCACAAGAAAGAAGTTTCTC
Gata6-luc/m3	GGGCATTAATTTTAGTGTGGCCGATGAGCCTAAGCG	CGCTTAGGCTCATCGGCCACACTAAAATTAATGCCC

## Supporting information

S1 Fig Confirmation of tissue-specific knockdown of Gata4 by gel electrophoresis. **A)** DNA was extracted from SHF, heart and tail tissues of *Gata4*^*fl/+*^*;Gli1-CreER*^*T2/+*^ mice and was tested for Cre-mediated knockdown of Gata4. **B)** DNA was extracted from heart and tail tissues of *Gata4*^*fl/+*^*;Nfatc1-Cre*^*+/-*^ mice and was tested for Cre-mediated knockdown of Gata4. **C)** DNA was extracted fromheart and tail tissues of *Gata4*^*fl/+*^*;Wnt1*-CreER^T/+^ mice and was tested for Cre-mediated knockdown of Gata4. **D)** DNA was extracted from SHF, heart and tail tissues of *Gata4*^*fl/+*^*;Osr1*-CreER^T/+^ mice and was tested for Cre-mediated knockdown of Gata4. **E)** DNA was extracted from SHF, heart and tail tissues of *Gata4*^*fl/+*^*;Mef2cAHF*::*Cre* mice and was tested for Cre-mediated knockdown of Gata4. **F)** DNA was extracted from heart and tail tissues of *Gata4*^*fl/+*^*;Tnt-Cre*^*+/-*^ mice and was tested for Cre-mediated knockdown of Gata4. The following primers were used: Cre forward: 5′-TCGACCAGGTTCGTTC ACTCATGG-3′; Cre reverse: 5′-CAGGCTAAGTGCCTTCTCTACACC-3′; Gata4-WT/flox forward: 5′-ACCCTGGAAGAC ACCCCAATCTCGG-3′; Gata4-del forward: 5′-TGTCATTCTTCGCTGGAGCCGC-3′; Gata4 reverse: 5′-TCCATGAGAC CCCAGAGTGTGCCTGA-3′. The size of Cre product is ∼220 bp. The Gata4 wild type and Gata4-flox products are ∼510 bp and 530 bp in size, respectively. The size of the Gata4-del product is ∼300 bp. **G)** Realtime-PCR results for Gata4 expression in the SHF of the *Gata4*^*fl/+*^*;Gli1-CreER*^*T2/+*^ versus the *Gata4*^*fl/+*^ embryos at E9.5 (TMX at E7.5 and E8.5). Data is presented as Mean±SEM, n = 6, ***P < 0.001 compared with the expression in *Gata4*^*fl/+*^ embryos. The forward primer used is 5’-GAAGAGATGCGCCCCATCAA-3’ and the reverse primer used is 5’- GCAGACAGCACTGGATGGAT-3’.
(TIF)
