# Supplementary figures and images for: Gata4 regulates hedgehog signaling and Gata6 expression for outflow tract development
Source: PLoS Genet. 2019 May 23;15(5):e1007711. doi: 10.1371/journal.pgen.1007711 (PMC6550424; doi:10.1371/journal.pgen.1007711)

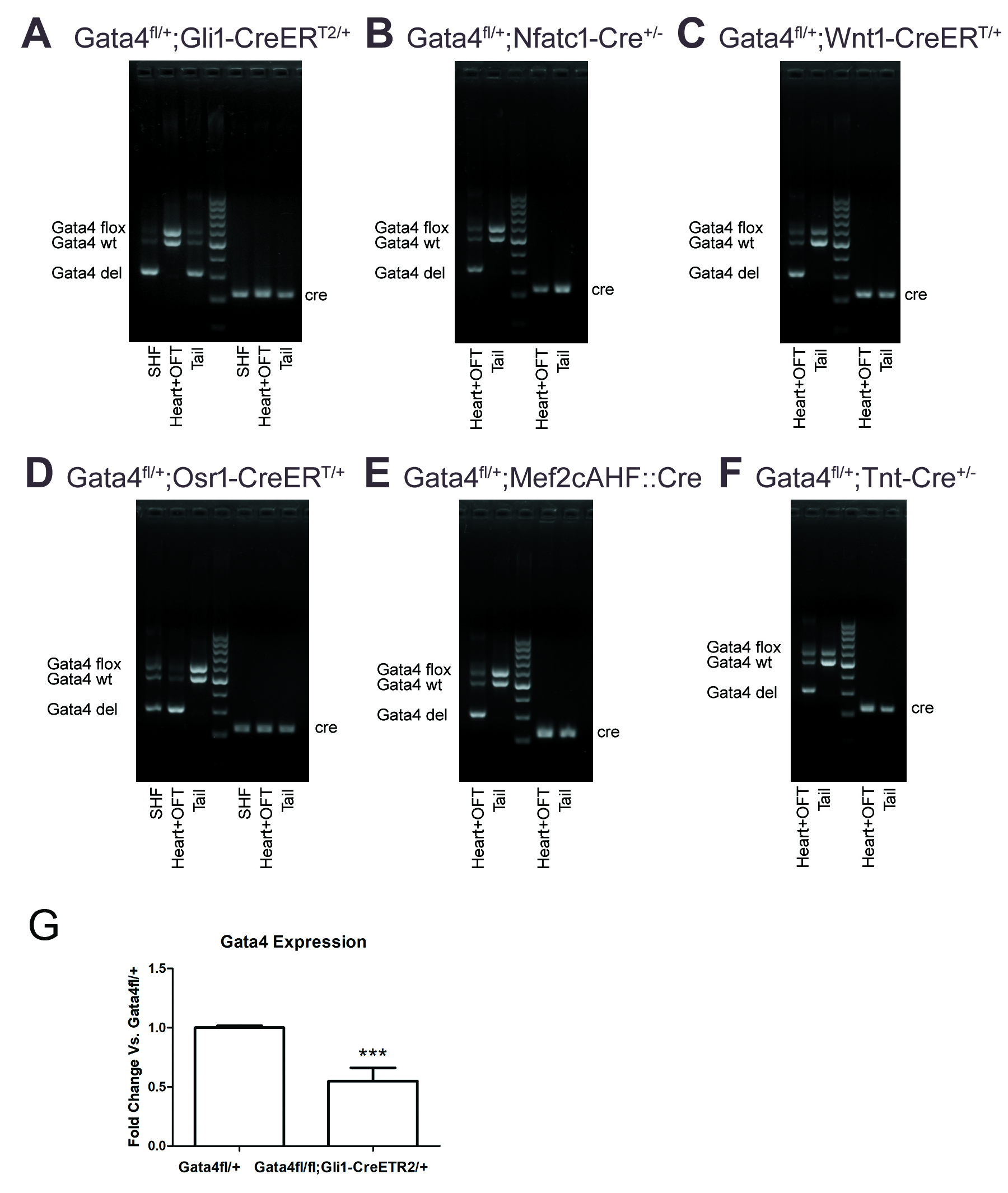

Supplement: S1 Fig — A) DNA was extracted from SHF, heart and tail tissues of Gata4fl/+;Gli1-CreERT2/+ mice and was tested for Cre-mediated knockdown of Gata4. B) DNA was extracted from heart and tail tissues of Gata4fl/+;Nfatc1-Cre+/- mice and was tested for Cre-mediated knockdown of Gata4. C) DNA was extracted fromheart and tail tissues of Gata4fl/+;Wnt1-CreERT/+ mice and was tested for Cre-mediated knockdown of Gata4. D) DNA was extracted from SHF, heart and tail tissues of Gata4fl/+;Osr1-CreERT/+ mice and was tested for Cre-mediated knockdown of Gata4. E) DNA was extracted from SHF, heart and tail tissues of Gata4fl/+;Mef2cAHF::Cre mice and was tested for Cre-mediated knockdown of Gata4. F) DNA was extracted from heart and tail tissues of Gata4fl/+;Tnt-Cre+/- mice and was tested for Cre-mediated knockdown of Gata4. The following primers were used: Cre forward: 5′-TCGACCAGGTTCGTTC ACTCATGG-3′; Cre reverse: 5′-CAGGCTAAGTGCCTTCTCTACACC-3′; Gata4-WT/flox forward: 5′-ACCCTGGAAGAC ACCCCAATCTCGG-3′; Gata4-del forward: 5′-TGTCATTCTTCGCTGGAGCCGC-3′; Gata4 reverse: 5′-TCCATGAGAC CCCAGAGTGTGCCTGA-3′. The size of Cre product is ∼220 bp. The Gata4 wild type and Gata4-flox products are ∼510 bp and 530 bp in size, respectively. The size of the Gata4-del product is ∼300 bp. G) Realtime-PCR results for Gata4 expression in the SHF of the Gata4fl/+;Gli1-CreERT2/+ versus the Gata4fl/+ embryos at E9.5 (TMX at E7.5 and E8.5). Data is presented as Mean±SEM, n = 6, ***P < 0.001 compared with the expression in Gata4fl/+ embryos. The forward primer used is 5’-GAAGAGATGCGCCCCATCAA-3’ and the reverse primer used is 5’- GCAGACAGCACTGGATGGAT-3’. (TIF) [file pgen.1007711.s001.tif]
